# Jacobsen Syndrome with Hypoplastic Left Heart Syndrome: Outcome after Cardiac Transplantation

**DOI:** 10.3390/jcdd10010008

**Published:** 2022-12-24

**Authors:** Federica Ferrigno, Alessio Franceschini, Richard Kirk, Antonio Amodeo

**Affiliations:** 1 The School of Pediatrics, University of Rome “Tor Vergata”, 00133 Rome, Italy; 2 Department of Cardiosurgery, Cardiology, Heart and Lung Transplant, Bambino Gesù Children’s Hospital, IRCSS, 00165 Rome, Italy; 3 Mechanical Circulatory Support Unit, Department of Cardiosurgery, Cardiology, Heart and Lung Transplant, Bambino Gesù Children’s Hospital, IRCSS, 00165 Rome, Italy

**Keywords:** Jacobsen syndrome, hypoplastic left heart syndrome, heart transplant, Norwood procedure, pediatric heart transplantation

## Abstract

Jacobsen syndrome (JS) is a rare syndrome caused by a deletion of chromosome 11q. We report a patient with JS and hypoplastic left heart syndrome (HLHS) who required cardiac transplantation. She had many of the recognized morphological features in addition to immunological (lymphopenia) and hematological (thrombocytopenia) issues. The patient underwent a Norwood procedure with a modified Blalock–Taussig shunt (MBTS) and subsequently a Glenn procedure at six months of age. She developed desaturation, with severe tricuspid regurgitation and right ventricular dysfunction, and underwent heart transplantation at 7 months of age. After the transplant, she was hospitalized several times for severe infections. The diagnosis of Jacobsen syndrome came 2 months after transplant. Now, 5 years post-transplant, she is in relatively good health—her heart is functioning normally, her hospitalization rate is getting lower, and her immunological profile is stable.

## 1. Introduction

Jacobsen syndrome (JS) is a rare contiguous gene syndrome caused by a deletion of chromosome 11q. The deletion size ranges from ~7 to 20 Mb, with the proximal breakpoint within or telomeric to sub-band 11q23.3, and the deletion extending usually to the telomere [1].

It was first described in 1973 by the Danish geneticist Petrea Jacobsen [2]. Since Dr Jacobsen’s first report, more than 200 cases of JS diagnosed after birth have been reported [3]. 

Phenotypic variability depends on the type and number of deleted genes [4] and includes multiple dysmorphic features [1], congenital heart disease [5], intellectual disability with mental and psychomotor retardation [6], physical growth retardation [7], visceral malformations and structural renal anomalies [8], neonatal thrombocytopenia and persistent platelet dysfunction (Paris–Trousseau syndrome) [9], and immunodeficiency (antibody deficiency and T-cell defects) [10]. 

Most of the deaths occur during the first 2 years of life for complications from congenital heart disease (CHD) [4], even though the life expectancy has been improved recentle, thanks to the successful management of complications such as bleeding and infections.

More than half of affected individuals have congenital heart disease (CHD), most of whom require surgical intervention. About one-third of patients with heart defects have a membranous ventricular septal defect (VSD), another third have left ventricular outflow tract defects with various degrees of hypoplasia or obstruction of the mitral valve, left ventricle, aortic valve, or aorta, and the final third have a variety of heart defects including double-outlet right ventricle, transposition of great arteries (TGA), atrio-ventricular septal defect (AVSD), secundum atrial septal defect (ASD), dextrocardia, aberrant right subclavian artery, patent ductus arteriosus (PDA), persistent left superior vena cava, tricuspid atresia, type B interrupted aortic arch (IAA), truncus arteriosus, and pulmonary valve stenosis (PVS) [5].

Hypoplastic left heart syndrome (HLHS), one of the most severe congenital heart malformations, occurs in 5% to 10% of patients with JS (compared to 0.02% in the general population) [11]. This percentage is also significantly higher than the occurrence in any other chromosomal disorders [12]. 

## 2. Patient

**Figure 1:**
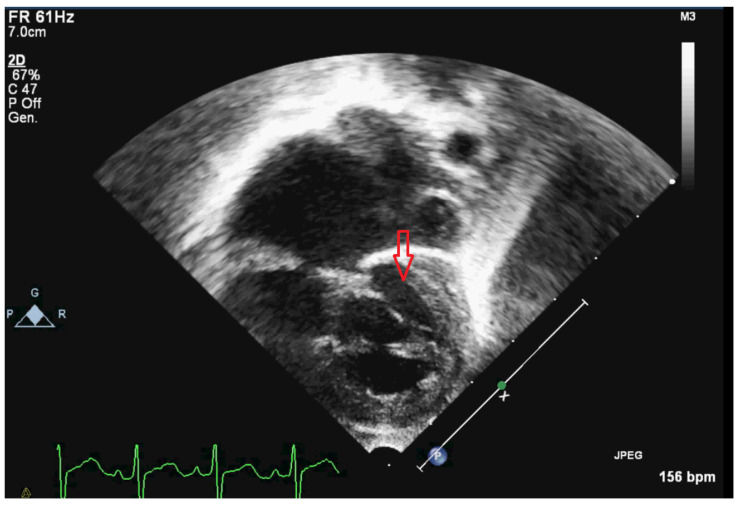
Apical four chambers view showing the left “slit like” ventricle and the atresia of the mitral valve plane. The arrow points at the left ventricle.

A Caucasian girl, born from a spontaneous conception, was delivered at 39 weeks by caesarian section for a prenatal diagnosis of HLHS with mitral-aortic atresia (see Figure 1). Her birth weight was 2.6 kg and the Apgar 8–9. The phenotype was characterized by macrocephaly, right choanal atresia, hypertelorism, flat nasal root, hypoplastic nose, and long fingers of the hands and feet (see Table 1). 

Moreover, the blood tests performed during the first months of life always showed a constant reduction of white blood count (WBC) and platelet count. Since the phenotype and the heart condition were highly suggestive of a genetic condition, she underwent large spectrum genetic analysis with a microarray-based comparative genomic hybridisation (CGH array) technique.

During the fourth day of life, she underwent a Norwood procedure. She was then readmitted to the hospital at one month of life with ventricular dysfunction and tricuspid valve insufficiency, due to aortic coarctation, and was managed with inotropic therapy. 

At two and a half months, she developed recoarctation and underwent end to end coarctectomy which was complicated by methicillin sensitive staphylococcus aureus (MSSA) sepsis, from which she recovered. 

Two months later, a Da Silva tricuspid valvuloplasty was performed for the tricuspid valve insufficiency. The post-surgery course was characterized by persistent severe tricuspid valve insufficiency requiring mechanical ventilation and continuous infusion of inotropes and diuretics. At six months of life, she underwent a bidirectional Glenn shunt and redo tricuspid valvuloplasty. The post-operative period was characterized by severe desaturation requiring a left jugular-carotid fistula. Following assessment, she was listed for cardiac transplantation and she was transplanted at seven months of life. Her post-operative immunosuppression was ciclosporin alone—cell cycle inhibitors were not used due to her neutropenia. Two weeks after transplant, she was diagnosed with septic shock with positive blood cultures for Klebsiella pneumoniae with extended-spectrum beta-lactamase (ESBL) and Escherichia coli, requiring antibiotic therapy. At this time echocardiography showed severe right ventricular dysfunction, presumed due to rejection, and she was pulsed with methylprednisolone. 

Due to multiple infectious events post-transplant, she started prophylaxis with antibiotic and antifungal drugs. The results of the CGH array became available, highlighting a 10.2 MB deletion of the terminal portion of 11q thus confirming the already suggestive clinical evidence of Jacobsen syndrome.

Five months after the transplantation, she was hospitalized for acute respiratory failure due to respiratory syncytial virus, rhinovirus, and bocavirus infections (detected by a nasopharyngeal aspirate) and required respiratory support with continuous positive pressure ventilation and high-flow nasal cannula. She was also given intravenous immunoglobulin infusions. During this episode, she was fed by a nasogastric tube because of the suspicion of aspiration and her refusal to feed orally, and subsequently underwent a percutaneous endoscopic gastrostomy. The respiratory situation was complicated further by a rotavirus infection that, along with the worsening of the respiratory function, required another hospitalization.

Five-and-a-half years after the transplantation, she now attends kindergarten. No other major infectious episodes have been reported.

She remains on immunosuppressive treatment with ciclosporin alone; she has normal cardiac function and her only recent intervention was a stent for stenosis of the superior vena cava, followed by warfarin therapy. Her last white blood cell count showed 8170/uL, with 440/uL lymphocytes, and 134,000 platelets/uL. A recent blood sample confirmed the immunological phenotype characterized by lack of CD4+ T helper lymphocytes.

## 3. Discussion

This case report is based on the management of HLHS in a patient with multiple complications due to JS. Both procedures, Norwood and transplantation, were particularly challenging due to the comorbidities.

Considering that the management of HLHS is mostly surgical, the first thing considered was the presence of thrombocytopenia. Moreover, in JS, platelet dysfunction is often associated with low platelets count [13]. At the time our patient required surgical interventions, the JS diagnosis was not known and so the platelet function was not tested. The surgical procedures were covered by platelet transfusion and were free from major bleeding events.

Another consideration was that the Norwood procedure usually requires antithrombotic therapy to maintain shunt patency and avoid thrombotic complications [14]. The American College of Chest Physicians Evidence Based Clinical Practice Guidelines for Antithrombotic Therapy and the Prevention of Thrombosis (9th ed.) recommends that newborns and children undergoing the MBTS procedure should receive intraoperative unfractionated heparin therapy (grade 2C), and either aspirin or no antithrombotic therapy, as compared to prolonged low-molecular-weight heparin (LMWH) or vitamin K antagonists postoperatively (grade 2C) [15]. Despite the persistent thrombocytopenia, she did not require maintenance platelet transfusions and she was treated with aspirin without any adverse effects. Furthermore, after the stent placement in the superior vena cava, she was treated for three months with warfarin, and no significant bleeding was reported.

Another feature of our patient’s complicated management was related to the immunological phenotype. Our patient was subjected to numerous blood test to detect the trend of the leukocyte values and the most affected subpopulation. Despite the leukopenia, she only had one significant infectious episode prior to transplantation.

During the hospitalization that led to the transplant, she was monitored closely for 9 months while she underwent 96 blood samples that showed an average total WBC number of 6446/uL 6000–17,500/uL, an average number of neutrophils of 4938/uL, an average number of lymphocytes of 872/uL, and an average number of platelets of 114,875/uL (see Table 2) [16]. 

After the lymphopenia was obviously persistent, the lymphocyte subpopulations were identified and it was found out that the population that was lacking the most was CD19+ Pan B. Then her immunoglobulin levels were screened, so it was evident that they were low as well, when compared to age-corrected values. 

The immunological assay of our patient could have been an important issue to consider during the first hospitalizations; in fact, our patient went through a lot of infectious complications that could have been prevented by the wise use of antibiotics. Moreover, the immunological assay represented an important item to consider before and after the transplant.

Generally, the major problem after the solid organ transplantation is the immunosuppressive drug to use to avoid rejection. In a patient with an underlying leukopenia, this choice is even more difficult. 

Since there are no published reports of transplantation in patients with JS, we can look at outcomes after heart transplantation in patients with other syndromic conditions associated with immunodepression. For example, patients with Barth Syndrome (BS) are usually affected by cardioskeletal myopathy and neutropenia. BS related neutropenia is associated with increased risk of sepsis, which is augmented by post-transplant immunosuppression. Nonetheless, a 2021 study [17] showed no difference in post-transplant survival and in freedom from important post-transplant complications, including infections, between individuals with BS who undergo transplant and non-BS transplant recipients with dilated cardiomyopathy. 

Our patient was treated with thymoglobulin during the induction phase of immunosuppression. The use of thymoglobulin could have been a problem in our patient, because of its additional adverse effect on platelets; in fact, it may cause thrombocytopenia beyond T-cell depletion [18]. Fortunately, our patient did not experience any of these effects. For maintenance, she started therapy with Ciclosporin, a calcineurin inhibitor, that is frequently used in organ transplants to prevent rejection, as it greatly improves the survival rates of patients after solid-organ transplantation [19]. By inhibiting calcium-dependent serine phosphatase calcineurin activity, ciclosporin interferes with the production and release of interleukin-2 (IL-2), a cytokine that is required for T-cell activation and proliferation [20,21]. Immunosuppression post-transplant generally incorporates a second agent as a cell cycle inhibitor such as Azathioprine or Mycophenolate mofetil (MMF)[22], but as these depress the WCC they were not used in our patient. 

After two weeks post-transplant, she had an episode of septic shock due to K. pneumoniae ESBL and E. coli. In view of this and her other frequent infectious episodes, prophylaxis with trimethoprim-sulfamethoxazole was commenced to good effect.

The immunological workup of our patient after the transplant and during the therapy with Cyclosporine determined a change in leucocyte subpopulation, from lacking CD19+ Pan B cells, to lacking CD4+ T Helper cells.

The change in the immunological phenotype may be compatible with the use of thymoglobulin, that, as reported in the literature, often causes T-cell lymphopenia with delayed reconstitution [23]. Unfortunately, no further evaluation regarding her immunological status was assessed.

Given the syndrome and the comorbidities, it is also worth mentioning that there was not any difficulty in managing the patient airway. In particular, the process of intubation and extubation was not reported as a problem during the multiple procedures she underwent or her multiple hospitalizations in the ICU.

## 4. Conclusions

The patient is now 6 years old, is well, and is attending kindergarten. This case demonstrates that even when detailed genetic knowledge and ramifications of a syndrome are not available at the time major intervention decisions need to be made, a successful outcome can still be achieved. Her post-transplant course has not been dissimilar to many non-JS children and indeed most syndromic children do well after transplantation as a recent study demonstrated, showing no difference concerning waitlist duration, survival to transplant, or post-transplant course **[24]**. Of course, our patient’s follow up needs to be strict, considering the possibility of an allograft vasculopathy, which is one of the major factors that affects long-term graft and patient survival after heart transplantation **[25]** and was described in a JS patient after cardiac transplantation during the 2010 11q Research and Resource Group Biennial Conference [26]. 

Whilst the Norwood procedure has been reported before [11], this is the first published report of heart transplantation in JS.

## Figures and Tables

**Table 1:** Patient Features.

Facies	Hypertelorism, Flat Nasal Root, Hypoplastic Nose
Cardiovascular defects	HLHS with mitral-aortic atresia (normal origin of the coronary arteries)
Neurological features	Intellectual disability
Immunological phenotype	Lymphopenia
Haematological features	Thrombocytopenia
Skeletal abnormalities	Macrocephaly
Limbs anomalies	Long fingers of the hands and feet
Other anomalies	Monolateral Choanal atresia

**Table 2:** Minimum and maximum values of our patient’s blood cells registered during the hospitalization, compared to normal values for age.

Type of Blood Cells	Minimum Value	Maximum Value	Normal Values for Age [16]
White cells	2380/uL	16,240/uL	6000–17,500/uL
Neutrophils	840/uL	10,100/uL	1500–8500/uL
Lymphocytes	872/uL	360/uL	4000–12,000/uL
Platelets	36,000/uL	277,000/uL	150,000–450,000/uL

## Data Availability

Not applicable.

## Abbreviations

ASD	atrial septal defect
AVSD	atrio-ventricular septal defect
BAV	bicuspid aortic valve
CGH	array microarray-based comparative genomic hybridisation
CHD	Congenital Heart Disease
HLHS	Hypoplastic Left Heart Syndrome
IAA	interrupted aortic arch
IL-2	interleukin-2
JS	Jacobsen Syndrome
LMWH	low-molecular-weight heparin
MBTS	Modified Blalock–Taussig Shunt
MMF	Mycophenolate mofetil
MSSA	Methicillin Sensitive Staphylococcus Aureus
PDA	patent ductus arteriosus
PVS	pulmonary valve stenosis
RV	right ventricle
TGA	transposition of great arteries
VSD	ventricular septal defect
WBC	white blood count
